# Deciphering the mechanism of action of 089, a compound impairing the fungal cell cycle

**DOI:** 10.1038/s41598-018-24341-y

**Published:** 2018-04-13

**Authors:** Irene Stefanini, Lisa Rizzetto, Damariz Rivero, Silvia Carbonell, Marta Gut, Simon Heath, Ivo G. Gut, Andrea Trabocchi, Antonio Guarna, Nagwa Ben Ghazzi, Paul Bowyer, Misha Kapushesky, Duccio Cavalieri

**Affiliations:** 1 CRI Centre for Research and Innovation, Fondazione Edmund Mach, San Michele all’Adige, Trento Italy; 2 Department of Biology, University of Florence, Florence, Italy; 3 CNAG-CRG, Centre for Genomic Regulation (CRG), Barcelona Institute of Science and Technology (BIST), Barcelona, Spain; 4 Universitat Pompeu Fabra (UPF), Barcelona, Spain; 5 Department of Chemistry “Ugo Schiff”, University of Florence, Florence, Italy; 6 Manchester Fungal Infection Group, Division of Infection Immunity and Respiratory Medicine, School of Biological Sciences, University of Manchester, Manchester, United Kingdom; 7 Manchester Academic Health Science Centre, University NHS Foundation Trust (Wythenshawe), Manchester, United Kingdom; 8 EMBL-EBI Wellcome Trust Genome Campus, Hinxton, United Kingdom; 9 Present Address: Division of Biomedical Sciences, Warwick Medical School, University of Warwick, Coventry, United Kingdom

## Abstract

Fungal infections represent an increasingly relevant clinical problem, primarily because of the increased survival of severely immune-compromised patients. Despite the availability of active and selective drugs and of well-established prophylaxis, classical antifungals are often ineffective as resistance is frequently observed. The quest for anti-fungal drugs with novel mechanisms of action is thus important. Here we show that a new compound, **089**, acts by arresting fungal cells in the G2 phase of the cell cycle through targeting of *SWE1*, a mechanism of action unexploited by current anti-fungal drugs. The cell cycle impairment also induces a modification of fungal cell morphology which makes fungal cells recognizable by immune cells. This new class of molecules holds promise to be a valuable source of novel antifungals, allowing the clearance of pathogenic fungi by both direct killing of the fungus and enhancing the recognition of the pathogen by the host immune system.

## Introduction

**Figure 1:**
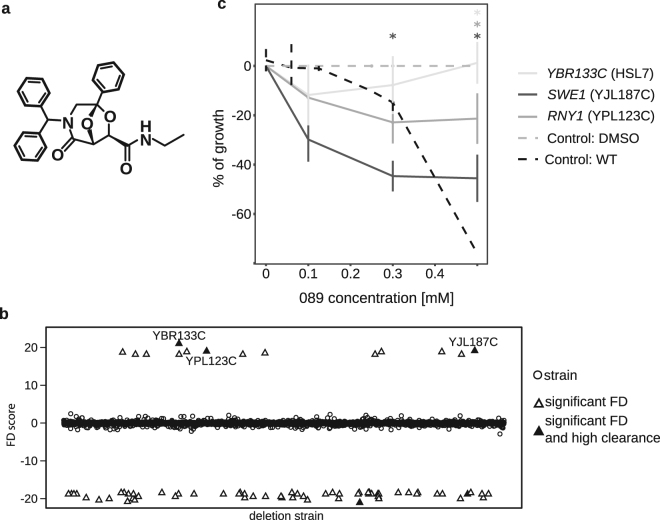
Profiling of the effect of **089**. **(a)** Chemical structure of **089**; (**b**) Fitness defect scores of the (HIP) heterozygous deletion strains treated with **089**. FD scores were calculated for each deletion strain as the difference of the abundance of the strain in the control compared to the treatment. Clearance, used as measure of the drug selectivity, was calculated as the difference of the FD of a given strain and the two closest FDs. Deletion strains having significant positive FD are potential targets of the compound; black triangles indicate the strains having significant FD and high clearance; (**c)** Effects of several concentrations of **089** on the growth of representative heterozygous strains selected based on the HIP results. The percentage of growth was calculated as the difference of the treated sample’s OD_600_ compared to the control OD_600_ (DMSO- treated deletion strain). Vertical bars indicate the standard deviation of three independent biological replicates. *Wilcoxon test p < 0.05 between the control and the strain indicated by the asterisk color, corresponding to the legend.

Over the past 20 years, the incidence of invasive fungal infections has increased^1^. While skin and nail infections have been estimated to affect ~25% of the total population worldwide, invasive fungal infections are uncommon, mainly affecting immuno-compromised patients who become more susceptible to fungal infections than healthy individuals^2^. Although invasive infections have an incidence much lower than superficial infections, the rate of mortality associated with invasive fungal infections is of particular concern, since it often exceeds 50% of the infected population^2^ and it is now estimated to be greater than mortality caused by malaria^3,4^. After the discovery of amphotericin B deoxycholate in the 1950s^5^, the discovery of new anti-fungal drugs has lagged behind the increasing need for their use. Amphotericin B is very effective for the treatment of a wide spectrum of fungal infections, but it has shown several side effects^6^. This led to the advent of new amphotericin B formulations and of a few new classes of anti-fungal, such as azoles and echinocandins^7^. Fungal infections are nowadays mainly treated with azole antifungals followed by candins. Yet the use of these new compounds and formulations is not sufficient to effectively treat fungal infections, mainly because of the insurgence of resistance in pathogenic fungi^8^. Thus, the quest for new anti-fungal drugs is urgent. For many years the budding yeast *Saccharomyces cerevisiae* has been chosen as one of the best cellular models for high-throughput identification of bioactive compounds and for their characterization. By using this approach, it is possible to identify new drugs with novel mechanisms of action. A new interest has grown in anti-fungal drugs able not only to kill the pathogenic fungus, but also to facilitate the host immune response^9,10^. Priming of the host immune response as an adjunct to conventional anti-fungal therapy represents a promising avenue to enhance the activity of antifungals. By exerting a direct pharmaco-therapeutic effect, fungal load may be directly reduced by the anti-fungal agent, thus providing time for the host to mount an effective immunologic program that will eradicate the invading pathogen without excessive inflammation. Several combined therapies where chemokines and cytokines are used in conjunction with classical antifungals have been proposed^11^. Voriconazole, fluconazole and echinocandins have been shown to stimulate the immune response through induction of regulatory chemokine and cytokine expression^12–14^. Here we show the mechanism of action of a new compound, **089** [(7R)-3-benzhydryl-2-oxo-5-phenyl-6,8-dioxa-3-aza-bicycle[3.2.1]octane-7-carboxyl-ethylamide, Fig. 1a]. This compound was identified in a chemical library encompassing small bi-cyclic molecules generated by combining tartaric acid and amino acids (BTAa)^15^. Our previous work showed that, the **089** compound had the highest anti-fungal activity against *Saccharomyces cerevisiae* strains (MIC, Minimal Inhibitory Concentration = 0.3 mM^15^) of the molecules in the library and was thus selected for further investigations. Here we show that this compound, by targeting the *SWE1* gene, conserved among pathogenic and non-pathogenic fungi, induces an impairment of the cell cycle with a consequent modification of cell morphology. This effect finally results in an increased recognition of treated fungi by immune cells, thus making the **089** chemical class a promising group of antifungals with potential for immune modulation.

## Results

### Identification of the mechanism of action of the new anti-fungal drug in the model fungus, *Saccharomyces cerevisiae*

To identify the molecular mechanisms underlying the effects induced by the anti-fungal drug **089** (Fig. 1a)^15^, we carried out a combination of analyses in the model yeast *S. cerevisiae* providing complementary information. Homozygous and heterozygous barcoded deletion collection sequencing was combined with transcriptional analysis and *in vivo* validation tests (Fig. S1). The heterozygous and homozygous barcoded deletion collections were generated in the model yeast *S. cerevisiae* and have been widely used to identify the mechanism of action of new and well-characterized compounds^16^. Each strain composing the deletion collection is deleted in a single gene, and the deletion is recognizable by the presence of specific DNA sequences (the “barcodes”)^17^. Hence, all the deletion strains can be pooled and treated at once with the compound of interest. By treating the heterozygous barcoded deletion collection (HIP, HapoInsufficiency Profiling) with **089**, we aimed to identify genes encoding proteins targeted by the treatment. In a heterozygous background, strains bearing a single copy of the target gene are more sensitive to the compound. Thus, a sub-lethal concentration of the anti-fungal is effective only against strains deleted in genes encoding proteins targeted by the anti-fungal. Because the deletion of a given gene could *per se* modify the growth ability of the strain, the results need to be compared to a control treatment (DMSO, the solvent used to dissolve the molecule of interest). Hence, we treated the deletion collections with a sub-lethal dose of **089** (0.2 mM, previously identified)^15^ or with a control treatment (DMSO), both treated for 20 generations at 27 °C in shaking culture and compared the relative abundances of each deletion strain. Fitness Defect (FD) scores were calculated for each deletion strain as the difference of the abundance of the strain in the control compared to that in the treatment (see materials and methods for further details). Aiming at the identification of targets, we searched for strains having positive FD in the heterozygous deletion collection (present at higher numbers in the control than in the treatment samples). 74 heterozygous deletion strains showed FD scores significantly different after the **089** treatment compared to the control (Fig. 1b). Among the strains having significantly different FD scores, only 14 strains showed positive FD scores, thus being candidates as **089** targets (Fig. 1b). To validate the results of the HIP analysis, we separately evaluated the effect of several **089** concentrations on the growth of the heterozygous deletion strains showing the highest significant FD and the highest clearance (the latter calculated as the difference in FD score between a given deletion strain and the deletion strains having the closest FD score) (Fig. 1c and Table S1). The YJL187C (*SWE1*) deletion strain was the most sensitive to the treatment, showing a high growth inhibition at the lowest tested concentration (0.1 mM, Mann-Whitney p < 0.05, Fig. 1c), three folds lower than the MIC evaluated for the wild-type strain. The morphogenesis checkpoint kinase Swe1p is one of the key regulators of the yeast cell cycle which regulates the G2/M transition and several other functions. This protein kinase acts by negatively regulating Cdc28p through phosphorylation, thus delaying mitosis and, when over-expressed, leads to cell cycle arrest in the G2 phase^18^. We also observed that the strain deleted in YBR133C (*HSL7*) showed a significant positive FD score in the HIP assay (Fig. 1b), but its killing by **089** was not confirmed in the single-strain assay (Fig. 1c). Hsl7p blocks the mitotic exit by means of Swe1p inhibition (by phosphorylation) and prevents Swe1p accumulation and ability to inhibit the cyclin complex composed of Cdc28p, Clb1p and Clb2p^19^.

### Homozygous deletion profiling analysis

**Figure 2:**
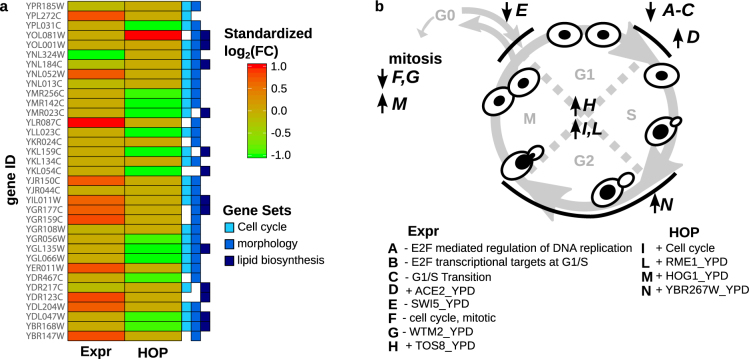
Effects of **089** on *Saccharomyces cerevisiae* cell cycle as indicated by HOP and expression analyses. (**a**) heatmap showing differentially expressed (microarray expression analysis) or differentially represented (HOP -Homozygous Profiling- analysis) genes involved in cell cycle, morphology and lipid biosynthesis. Only genes significantly differentially represented/expressed, belonging to one of the three gene sets and showing a log2(fold change) value higher than 1 or lower than −1 are shown. For graphical representation, values were scaled between −1 and 1. FC = Fold Change, ratio; (**b**) the circle summarizes the **089** effects, found by means of HOP and expression analysis, on the *S. cerevisiae* cell cycle. Gene sets significantly differentially represented after the treatment with **089** are reported as capital letters in the scheme and written in the extended form in the legend. In the scheme, black arrows pointing up indicate an over represented gene set, black arrows pointing down indicate under represented gene sets (Fisher Exact Test p-value < 0.05). In the legend, plus and minus indicate over- and under- represented gene sets.

The treatment of the homozygous barcoded deletion collection (HOP, HOmozygous deletion Profiling) with a sub-lethal concentration of the investigated compound (0.2 mM) allows the identification of pathways targeted by the treatment or involved in the response to it. Aiming at the assessment of the cell response to the treatment, we treated the homozygous deletion collection as the heterozygous deletion collection (see previous paragraph) and searched for strains having significantly different FD scores between the treatment with **089** and the control (DMSO). As expected, the number of deletion strains having significant FDs identified after the treatment with **089** was higher in the HOP compared to the HIP assay (92 strains the first, 74 the latter, Table S1 and Fig. 1b). The list of FDs significantly differing in the treatment with **089** compared to the control (Table S2) was analyzed by means of pathway signature analysis, which allows determination of gene sets (either pathways or sets of genes regulated by the same transcription factor) that are differentially represented in the compared conditions^20^. Positive HOP FD scores correspond to strains deleted in genes involved in the response to the treatment and negative HOP FD scores indicate strains deleted in genes (or involved in pathways) targeted by the treatment. Hence, the same interpretation can be used for pathway analysis. All the differentially represented gene sets were over-represented (Table S2). Several differentially represented gene sets were related to the cell cycle (Fig. 2a), thus supporting the targeting of Swe1p by the molecule: the cell cycle as described in the KEGG database^21^ and the genes regulated by Hog1p and Rme1p transcription factors^22^. In addition, gene sets related to the cell morphology (associated to YBR627W, Gat3p, Dot6p, Hms1p transcription factors’ activity, Fig. 2a) were over represented in HOP analysis.

### Pathway analysis on *S. cerevisiae* gene expression

To further contextualize the HIP and HOP results, the effect of the compound on the model yeast *S. cerevisiae* was analyzed at the molecular level through gene expression microarray analysis (Table S3). To accomplish this, the *S. cerevisiae* haploid laboratory strain BY4742 was treated with a sub-lethal concentration of **089** (0.2 mM), for 4 hours at 27 °C in shaking and compared the resulting transcriptional profile with the control transcriptional profile (cells treated with DMSO). Pathway signature analysis^20^ was carried out on the transcriptional profile induced by the treatment (Table S3). The results indicated impairment of the cell cycle, further supporting the targeting of Swe1p by **089** (Fig. 2b). Pathway analysis revealed that **089** allowed cells to proceed through the G1 and S phases and caused a cell arrest in the G2 phase (under-expression of genes related to the G1/S transition, to the E2F transcriptional targets at G1/S, and to the mitotic cell cycle pathways, Fig. 2b). In addition, a modification of cell morphology pathways was observable through pathway analysis, indicating a decrease in glycan structures (under-expression of their biosynthesis and over-expression of their degradation) and the inhibition of pseudo-hyphal filamentation or flocculation (genes controlled by Hms1p and Sfl1p). Furthermore, genes related to sphingolipid metabolism were also found over-expressed in response to the treatment with **089** (Table S3). The low number of gene sets differentially represented after **089** treatment and the fact that they are associated to only three main classes (cell cycle, morphology and lipid metabolism) is an indication of the molecule’s selectivity. To assess the novelty of the mechanism of action of the new compound **089**, we compared the **089** HIPHOP profile with those of 3356 compounds published by Lee and coworkers^16^. The clustering of HIPHOP profiles and the following profiles grouping defined through the dynamic branch cutting method highlighted a **089** HIPHOP similarity to 27 HIPHOP profiles of the Lee database (Fig. S2). Embedded in the sub-cluster encompassing the **089** HIPHOP, there was a group of treatments identified to induce a modification of tryptophan biosynthesis and mitochondrial translation (Table S4)^16^. In addition, only two of the **089** co-clustering treatments have a known target (*RER2* for both), coding a cis-prenyltransferase involved in dolichol synthesis and participating in endoplasmic reticulum protein sorting^16^.

### Validation and phenotypic effects of Swe1p targeting in *S. cerevisiae* by the **089** compound

**Figure 3:**
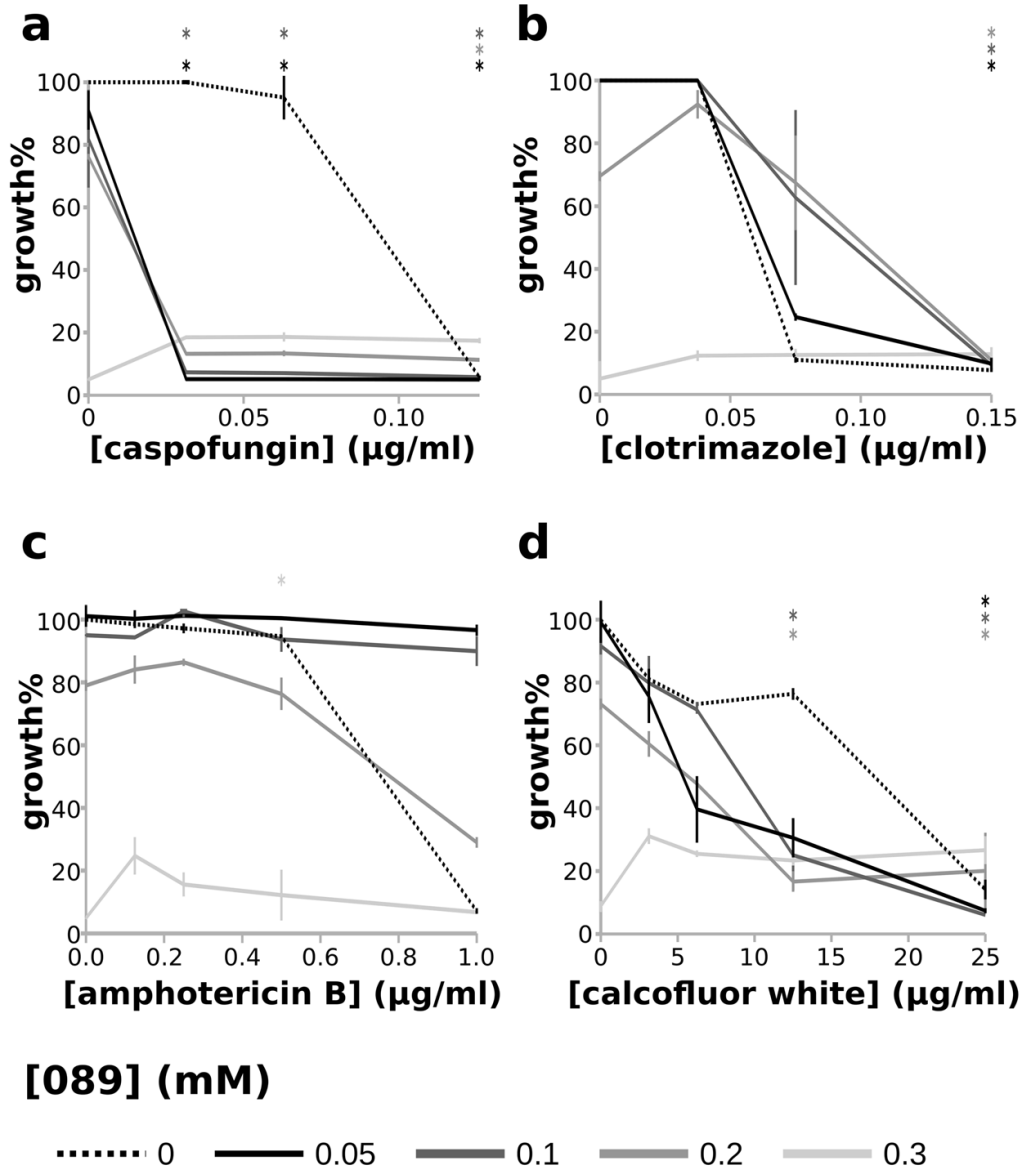
*Effects on S. cerevisiae* growth of **089** in combination with compounds known to perturb the cell wall. BY4742 *S. cerevisiae* cells were treated with lethal (MIC, 0.3 mM) and sub-lethal (0.2 mM, 0.1 mM, 0.05 mM) concentrations of **089** combined with lethal and sub-lethal concentrations of caspofungin (**a**) clotrimazole (**b**) amphotericin B (**c**) and calcofluor white (**d**). Treatments were carried out in YPD for 24 hours at 28 °C with shaking. Growth was quantified by measuring the OD_600_ (Optical Density at 600 nm). For each treatment, growth % was calculated as the percentage of OD compared to the control (treated with DMSO). 2-tailed t-test was performed to evaluate differences between the effect of each combined treatment and the effect of the **089** lethal concentration (0.3 mM) and p-values were FDR corrected. *Treatments not significantly changing compared to the **089** lethal concentration (FDR > 0.05).

HIP, HOP, and transcriptional analyses suggested that **089** impairs the *S. cerevisiae* cell cycle and arrests cells in the G2 phase. To validate this observation, we used flow-cytometry to quantify the DNA content of cells treated with **089** compared to the DNA content of control cells (treated with DMSO). To avoid the inclusion of budding cells in the analysis which would skew the quantification of single-cell DNA content, the DNA content was estimated only for cells showing forward and side scatter values similar to those of a culture of the BY4742 haploid strain. Cells of the haploid laboratory strain BY4742 were treated for 4 hours with a lethal concentration of **089** (0.3 mM) and their DNA content was determined by Propidium Iodide labeling in comparison to haploid BY4742 untreated cells and diploid cells obtained by crossing the BY4742 and W303 laboratory strains (Table 1). A significantly increased number of cells bearing a doubled content of DNA (characterizing the G2/M phase) was observed after treatment with **089** compared to DMSO (n = 3, 2-tailed t-test p < 0.01). Thus, flow-cytometry analysis confirmed that cells treated with the **089** molecule accumulated in the G2/M phase (Fig. S3a). The arrest of the cell cycle in the G2/M phase due to an altered Swe1p activity has previously been associated with reduced fidelity in bipolar budding^23^ and to an altered bud morphology^24^. In accordance with this, cells treated for 4 hours with 0.3 mM **089** showed an abnormal bud morphology: they elaborated multiple simultaneous budding events, indicating either that daughter cells do not separate after the budding or that multiple daughter cells emerge at the same time (Fig. S3b). A modification of cell morphology was also observed by means of pathway analysis on the transcriptional profiles induced by **089** treatment (Table S3), and in particular a decrease in glycan structures and the inhibition of pseudo-hyphal filamentation or flocculation were observed. To further support these observations, we evaluated the effects of combined treatments of *S. cerevisiae* cells with **089** and compounds known to modify cell wall composition or structure. We tested the combined effect of **089** with caspofungin (an echinocandin, inhibiting β-glucan biosynthesis), clotrimazole (an azole, inhibiting ergosterol biosynthesis), amphotericin B (forming pores on the cell membrane), and calcofluor white (binding chitin). BY4742 cells were treated for 24 hours at 28 °C with a lethal concentration of **089** (0.3 mM) and sub-lethal **089** concentrations (0.2 mM, 0.1 mM, and 0.05 mM) alone or combined with lethal or sub-lethal concentrations of the cell wall-affecting compounds (Fig. 3). Notably, the combination of the lowest tested **089** concentration (0.05 mM) and the lowest tested caspofungin concentration (0.0315 µg/ml) induced an effect statistically similar to the effect induced by the treatment with the lethal **089** concentration (0.3 mM; 2-tailed t-test, FDR > 0.05). A similar effect was observed in the **089**-clotrimazole combined treatments. Nevertheless, the **089**-clotrimazole combination was as lethal as 0.3 mM **089** only in the presence of 1.5 µg/ml clotrimazole (corresponding to the MIC). On the other hand, combined treatment with amphotericin B resulted in *i*) a reduction of the lethal effect of 0.3 mM **089**, *ii*) a slight decrease of the cell growth in presence of 0.2 mM **089** or *iii*) no variations in the effects induced by lower **089** concentrations (Fig. 3). The combined treatment with calcofluor white had a similar effect, reducing the 0.3 mM **089** lethality and slightly increasing the effect of lower **089** concentrations. The changes in cell wall composition were also assessed by means of calcofluor white staining. BY4742 *S. cerevisiae* cells were inoculated in YPD supplemented with either 0.3 mM **089** or DMSO and treated for 4 hours at 28 °C with shaking. The cells treated with **089** showed a reduced labeling with calcofluor white compared to the control, especially when considering emerging new cells (Fig. S5).

**Table 1:** Yeast, and fungal strains and mammalian cell lines used in this study.

Strain	Species	Genotype	Characteristics	Used in the assay:
BY4742	*S. cerevisiae*	MATα his3Δ1 leu2Δ0 lys2Δ0	Laboratory/reference strain	1. background for heterozygous and homozygous deletion collections used for HIP and HOP analyses.
				2. Transcriptomic analysis (microarray hybridization)
				3. phenotype validation (DNA content, budding morphology, phospholypid quantification, DC-binding)
BY4742 *SWE1*::KANMX4	*S. cerevisiae*	MATα his3Δ1 leu2Δ0 lys2Δ0 SWE1::KANMX4	Laboratory/reference strain	Target validation
BY4742 *HSL7*::KANMX4	*S. cerevisiae*	MATα his3Δ1 leu2Δ0 lys2Δ0 HSL7::KANMX4	Laboratory/reference strain	Target validation
BY4742 *RNY1*::KANMX4	*S. cerevisiae*	MATα his3Δ1 leu2Δ0 lys2Δ0 RNY1::KANMX4	Laboratory/reference strain	Target validation
BY4742xW303	*S. cerevisiae*	*leu2Δ0/leu2-3,112 TRP1/trp1-1 can1-100 ura3Δ0/ura3-1 ADE2/ade2-1 his3Δ1/his3-11,15 LYS2/lys2Δ0 [phi* ^+^ *]*	Laboratory/reference strain	Reference for DNA content determination
YUC22	*C. glabrata*	Wild-type	Clinical isolate	MIC evaluation in *Candida* spp.
SC5314 (*ATCC* MYA-2876)	*C. albicans*	Wild-type	Laboratory/reference strain	MIC evaluation in *Candida* spp.
A1160	*A. fumigatus*	*akuB* (*KU80*)-delta *pyrG1 MAT1-1*	Laboratory/reference strain	1. MIC evaluation in *Aspergillus* spp.
				2. transcriptomic analysis (RNAsequencing)
CYP51KO	*A. fumigatus*	*(cyp51A)-delta akuB* (*KU80*)-delta *pyrG1 MAT1-1*	Azole hyper-sensitive	MIC evaluation in *Aspergillus* spp.
RFLS58	*A. fumigatus*	Wild-type	Clinical isolate resistant to azoles	MIC evaluation in *Aspergillus* spp.
K562 (ATCC CCL-243)	*H. sapiens*	Wild type	Immortalised myelogenous leukemia	**089** toxicity on mammalian cells

Swe1p has also been recently shown to positively regulate sphingolipid biosynthesis by phosphorylating and inhibiting Orm proteins^25^. Also in this context, the targeting of *SWE1* by **089** was confirmed by the observation that, after a 24 hours treatment with 0.3 mM **089**, ceramides accumulated over free fatty acids, indicating an induction of *de novo* sphingolipid biosynthesis (Fig. S3c). We note that this effect was also observed at the pathway level through transcriptional analysis (Table S3).

### The **089** anti-fungal activity against pathogenic fungi

**Figure 4:**
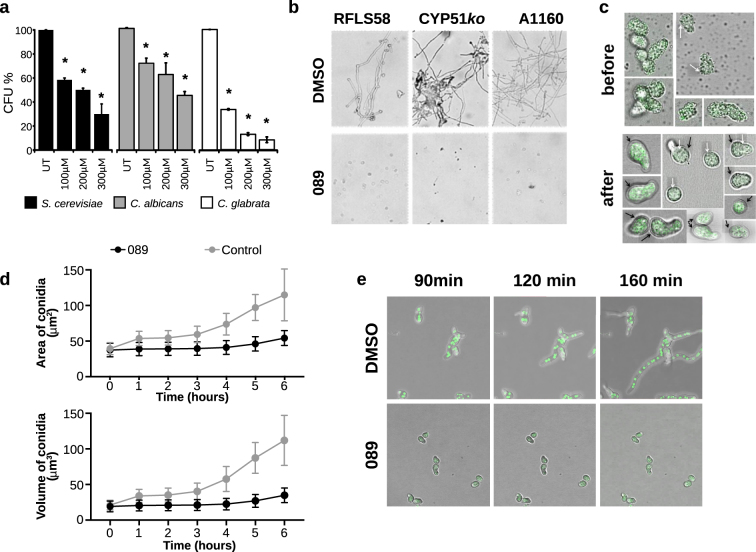
The effects of **089** on pathogenic fungi. (**a**) Effects of **089** on *Candida* spp. cells; 5 × 10^6^ cells/ml of *S. cerevisiae* (black bars), *Candida albicans* (grey bars) and *C. glabrata* (white bars) cells were treated with several concentrations of **089** or DMSO as control (UT). After 4 hours of treatment in YPD at 30 °C, 100 live cells were plated onto solid YPD and formed colonies were counted after at least 4 days. Values indicate Colony-Forming Units (CFU) average + SD (*n* = 3), ANOVA test was carried out, using Fisher as post-hoc test to compare treatments, *p < 0.0001 (**b**) Representative images of the **089** effects on hyphal formation. *Aspergillus fumigatus* spores (2.5 × 10^5^ spores/ml in RPMI 1640 medium + 2% glucose) were treated with serial dilution of **089** ranging from 1 mM to 25 µM were added in 96-well plates containing. The effects were microscopically inspected after 48 hours. The images show the effects of 0.4 mM **089** (concentration at which all the strains showed the phenotype) or DMSO as control. (**c**) Internalization of **089** drug granules within the fungal conidia/ germling was evaluated by labelling drug granules approximately 30 minutes post treatment. The localization of drug granules was assessed before and after washing twice the treated cells with PBS. Images were acquired before and after washing. White arrows refer to drug granules, black arrows refer to conidia/ germling cell wall. (**d**) Effects of **089** on *A. fumigatus* conidia germination. The volume and area of conidia treated with 0.4 M **089** or with DMSO as control were measured using Imaris software (Bitplane, UK) of a confocal acquisition time lapse over 6 hours. (**e)** Effects of **089** on *A. fumigatus* cells growth – hyphal elongation. An A1160 *A. fumigatus* strain expressing the labelled Histone 1 protein (H1-sGFP) was used to evaluate the effect of the treatment on hyphal elongation, indicated by the formation of polynucleated cells. H1-sGFP conidia were grown in Sabouraud medium for 6 hours at 37 °C, then germlined cells were treated with 0.4 mM **089** or with DMSO as control. After 90 minutes treatments, images were acquired each 10 minutes.

The increasing emergence of drug-resistant pathogenic fungal strains led us to consider the potential of **089** to inhibit the growth of clinically important pathogens. Hence, we assessed the effect of the treatment with **089** at various concentrations (0.3 mM, 0.2 mM, 0.1 mM) on the survival of *Candida albicans* and *C. glabrata* cells treated for 4 hours in YPD at 28 °C with shaking. After 4 hours treatment, we calculated the percentage of surviving cells as described in methods section. Following this assessment 100 live cells were plated onto YPD agar medium. After 4 days the CFU % (Colony Forming Units %) was calculated as the number of colonies formed by treated cells compared to the number of colonies formed by control cells. Each concentration of **089** induced a significant decrease in CFU in all the assayed yeast species (ANOVA test, Fisher post-hoc, *p* < 0.00001), indicating that **089** was effective also against pathogenic yeasts (Fig. 4a). The effects of **089** were also investigated using three different strains of *Aspergillus fumigatus* (Fig. S5a): a wild-type strain, A1160, and two azole-resistant strains, CYP51KO (deleted in th*e cyp5*1A gene and azole hyper-sensitive) and RFLS58 (an azole resistant environmental isolate). *A. fumigatus* conidia were inoculated at 2.5 × 10^5^ spores/ml in MOPS-buffered RMPI 1640 medium supplemented with 2% glucose and several concentrations of **089** (serial dilutions from 1 mM to 0.025 mM) or DMSO as control. The treatment was carried out for 48 hours in static at 37 °C. At the end of the treatment, cultures were microscopically inspected to evaluate the cell survival and the cell morphology. The MIC (Minimum inhibitory concentration, the lowest concentration inducing fungal death) evaluated for azole-resistant strains CYP51ko was 1 mM, while for the A1160 reference strain was even higher (Fig. S5a). Nevertheless, a strong inhibition of the conidia germination induced by **089** was observable in all the tested strains (Fig. 4b). The MEC (Minimum Effective Concentration), considered as the minimal concentration inhibiting hyphae formation, differed among the tested *A. fumigatus* strains. The CYP51ko strain was the most sensitive (MEC = 0.3 mM) and the other azole-resistant strain (RFLS58) was the less sensitive to the treatment (MEC = 0.5 mM, Fig. S5a). To further characterize the effect of **089** on *A. fumigatus*, we first evaluated the internalization of the compound. We observed the localization of microscopic auto fluorescent drug particles before and after a PBS washing of treated cells (Fig. 4c). Before washing, drug granules were evident on the surface of conidia and germling cells excluding the view of cell contents, whereas after washing, the fluorescent compound was localised within the cell cytoplasm thus showing the ability of the compound to enter the cells (Fig. 4c). To further validate the inhibitory effect of **089** on *A. fumigatus* conidia germination, we measured swelling of A1160 *A. fumigatus* spores exposed to the compound (Fig. 4d). The area and volume of A1160 conidia treated with 0.4 mM **089** or with DMSO as control was measured in Sabouraud medium at 37 °C over a time course of 6 h. In the control treatment spores swelled evenly and continuously, but neither volume or area of conidia treated with **089** changed during the treatment (Fig. 4d). Treated spores did not germinate. (Fig. 4d). Since *A. fumigatus* often initiates infections in hyphal form, we also evaluated the effect of the compound on *A. fumigatus* hyphae (Fig. 4e). If the cell cycle inhibition induced by **089** observed in *S. cerevisiae* cells is conserved in *A. fumigatus*, fungal cells treated with the compound should not be able to grow and form multinucleate cells. Hence, we evaluated the number of nuclei in an A1160 strain expressing a GFP labelled Histone 1 protein (H1-sGFP). H1-sGFP treated with **089** compared to the control treatment (DMSO). Conidia were grown in Sabouraud media for 6 hours, allowing conidial germination. Afterwards, cells were treated with 0.4 mM **089** or with DMSO and images were acquired after 90 minutes every 10 minutes. The growth of hyphae treated with the **089** compound was clearly inhibited, as indicated by the fact that treated cells did not form elongated, polynucleated cells as in the control. Additionally, nuclei did not divide in cells treated with **089** (Fig. 4e and Supplementary Movie S1–S3).

### The effects of **089** on *Aspergillus fumigatus* at the molecular level

**Figure 5:**
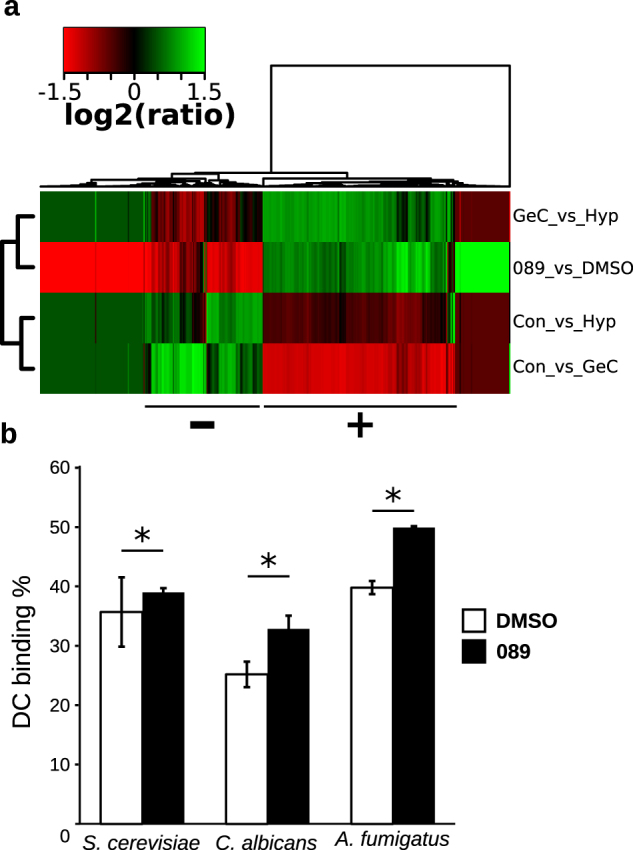
The effects of **089** on *Aspergillus* *fumigatus* and on the binding of dendritic cells. (**a**) Comparison of the transcriptional profile induced by **089** with the profiles of germinating conidia compared to hyphae (GeC_vs_Hyph), of conidia compared to hyphae (Con_vs_Hyph) and conidia compared to germinating conidia (Con_vs_GeC). Samples and genes distances were calculated with the Manhattan method and clustered with the Ward method. (**b**) Ability of dendritic cells (DC) to bind fungal cells treated with **089** or with DMSO (control). DC were differentiated from healthy donors PBMCs isolated monocytes and exposed to FITC-labelled fungi (stimuli:DC ratio 5:1) for 30 minutes at 37 °C. DCs were labelled with anti-CD11c-PE. The binding of DCs to fungi was measured by flow cytometry. *Mann-Whitney p < 0.0001.

The effects of **089** exposure on the *A. fumigatus* wild-type strain were investigated at the molecular level by means of RNA-sequencing analysis and compared to the transcriptional profile of a control culture (treated with DMSO). Conidia were inoculated at 2.5 × 10^5^ conidia/ml in MOPS-buffered RPMI 1640 + 2% glucose supplemented with either 0.1 mM **089** or an equal volume of DMSO. After 4 hours of static growth at 37 °C, mycelium was frozen in liquid nitrogen, ground to a fine powder then RNA was extracted, and Illumina sequenced. Genes whose expression significantly changed between the treated and the control cultures (FDR < 0.05) were identified as DEGs (Differentially Expressed Genes). We found 436 DEGs compared to the control. Of these, 209 were under-expressed (their transcripts are more abundant in control samples than in treated samples, 166 with log2ratio < −2) and 227 were over-expressed (their transcripts are more abundant in the treated samples than in the control samples, 95 with log2ratio > 2) (Table S5). Considering the over-expressed genes, we found an enrichment in Gene Ontologies (GO) related to iron starvation response, response to drug, iron and phosphate transmembrane transport, and secondary metabolite biosynthesis (Table S6). These processes are known to be involved in a feedback loop regulation of the MAPK cell wall integrity pathway which is also involved in the regulation of phenotypes such as radial growth and conidiation^26^, thus possibly explaining the observed inhibition of *A. fumigatus* spore germination. By inspecting the phenotypic effects of the treatments on *A. fumigatus* cultures we observed an evident impairment of hyphae formation (Fig. 4c). Hence, we wondered whether this effect was also observable at the molecular level. To evaluate this, we compared the transcriptional profile induced by **089** with a set of transcriptional profiles obtained in *A. fumigatus* cells at different stages of the asexual cycle^27^, namely conidia (con), germinating conidia (GermCon) and hyphae (Hyph). To generate this dataset, Hagiwara and colleagues^27^ obtained DEGs by comparing the genes’ expression levels in: *i*) *A. fumigatus* conidia against hyphae (Con_vs_Hyp), *ii*) conidia against germinating conidia (Con_vs_GeC), or *iii*) germinating conidia against hyphae (GeC_vs_Hyp). The transcriptional profile of cells treated with the **089** compound clustered with the GeC_vs_Hyp profile, obtained by comparing germinating spores against hyphae (Fig. 5a). Hence, this similarity supported what observed at the phenotypic level (Fig. 4c). We went further and searched for groups of genes whose expression level was similar in the **089** treatment and in the GeC_vs_Hyp comparison but was the opposite in the Con_vs_GeC and Con_vs_Hyp profiles (Fig. 5a). We found two relevant sub-groups of genes: the first, indicated with “+” in Fig. 5a, was composed by genes over-expressed in the **089** treatment and in the GeC_vs_Hyp profiles and under-expressed in the Con_vs_Hyp and Con_vs_GeC profiles; the second, indicated with “−” in Fig. 5a, encompassed genes under-expressed in **089** and GeC_vs_Hyp profiles and over-expressed in Con_vs_Hyp and Con_vs_GeC profiles. Notably, the first set of genes (overexpressed in **089** and GeC_vs_Hyp and under-expressed in Con_vs_Hyp and Con_vs_GeC), was enriched in GOs related to ribosomes and to rRNA processing, synthesis and modification (Fig. S5b).

The effects observed at the molecular and phenotypic levels, both suggesting an impairment of asexual cell cycle progression in *A. fumigatus* and correspond with the impairment of cell cycle through Swe1p targeting observed in *S. cerevisiae*. To translate the **089** target identified in *S. cerevisiae* in opportunistic fungal infections we looked at the presence of *SWE1* in common fungal pathogens. Both *Candida glabrata* and *C. albicans* bear orthologs of the candidate target *SWE1* (CAGL0K00693g and C1_10010C_A, respectively, Fig. S6). Swe1p in *C. albicans* was shown to be required for full virulence, but not for filamentous growth. *A. fumigatus* contains an uncharacterized protein, Afu2g07690, with the highest sequence similarity to the *S. cerevisiae* Swe1p (the phylogenetic tree of the fungal orthologues is shown in Fig. S6). Despite *SWE1* having two known homologs in humans (*WEE1* and *WEE2*), **089** showed a moderate toxicity on mammalian cells, with none of the **089** tested concentrations significantly reducing cell survival (Fig. S7).

### Effects of **089** on fungal recognition by dendritic cells

Several pathogenic fungi (i.e. *Candida* spp.) switch their phenotype to invade the host tissue, evade the host immune response and shift it towards anti-inflammatory response^28^. Since **089** was shown to alter fungal ability to form hyphae which are more easily recognized by the host immune system, we investigated whether this effect could in turn modify the recognition of treated fungal cells by immune cells. We thus compared the *in vitro* ability of human monocyte-derived dendritic cells (DCs) to recognize and bind **089** treated *S. cerevisiae*, *C. albicans* and *A. fumigatus* cells. Aiming at observing potential improvements in the receptor recognition of viable yeast and fungal cells *S. cerevisiae*, *C. albicans* and *A. fumigatus* cells were treated with sub-lethal concentrations of the **089** compound (0.2 mM) for 4 hours. *S. cerevisiae* and *C. albicans* cells were treated in YPD at 28 °C with shaking, *A. fumigatus* cells were treated in MOPS-buffered RPMI medium at 37 °C in static. After the treatment, yeast and fungal cells were FITC-labeled and their binding (or internalization) by DC was measured through flow-cytometry (see materials and methods section for further details). DCs were able to bind or internalize a higher percentage of both *C. albicans* and *A. fumigatus* cells treated with 0.3 mM **089**, compared to the same fungal cells exposed to DMSO only (Mann-Whitney p < 0.05, Fig. 5b).

## Discussion

The parallel utilization of several tools in the model yeast *S. cerevisiae* allowed the discovery of the mechanism of action of the new anti-fungal drug **089**. We took advantage of the heterozygous barcoded deletion strain collection to identify drug targets and to observe affected and response pathways as well as using transcriptional profiling to uncover effects of the drug at the expression level in the entire cell. The combination of these techniques gives a complete picture of the effects induced by **089** treatment. The restricted number of potential targets identified by mean of HIP analysis may predict a high level of selectivity of the molecule. In confirmation the pathway level analysis only indicated effects directly associated to *SWE1* targeting. The anti-fungal **089** acts on Swe1p, which controls cell cycle progression by delaying the G2/M transition through phosphorylation of Cdc28p. Both the phenotype and pathway analyses strongly support the Swe1p targeting by **089**. While the deletion of the identified **089** target *SWE1* induces an altered cell cycle kinetics with progression through the G2/M phase transition^29^, Swe1p over-expression induces cell arrest in G2/M (Fig. S3a)^30^. Hence, it could be hypothesized that **089** induces Swe1p activation. To note, the hypothesis that **089** activates Swe1p was further supported by the observation that the heterozygous *HSL7* gene deletion strain showed a significantly positive FD after treatment with **089** (Fig. 1b). One of the functions of Hsl7p is to clear Swe1p from the cytosol. Thus, in the *HSL*7 heterozygous deletion strain, Swe1p accumulates in the cytosol, resulting in an effect similar to the one induced by **089**. The arrest of the cell cycle in the G2/M phase caused by Swe1p over-expression has been associated to a reduced fidelity in bipolar budding^23^ and to an altered bud morphology^24^. The activation of Swe1p by **089** was further confirmed by the observation of the accumulation of ceramides over free fatty acids, indicating an induction of *de novo* sphingolipid biosynthesis (Fig. S3c). The potential counter-action of **089** and amphotericin B (especially at the **089** MIC, 0.3 mM) could be ascribed to the fact that amphotericin B acts by forming pores on the cell membrane and may be sensitive to the lipid/sphingolipid ratio. The observation that calcofluor white cell wall labeling of *S. cerevisiae* cells treated with **089** was reduced compared to the control treatment may suggest interplay between chitin synthesis and Swe1p, especially during early growth stages. Furthermore, the astonishingly high synergistic effect of **089** with caspofungin could be the result of a similar modification of the cell wall β-glucans content. Hence, the effect of **089** on Swe1p leads to a modification of cell morphology, observed both in *S. cerevisiae* and in *A. fumigatus*.

The HIPHOP profile induced by **089** treatment was similar to the HIPHOP profile induced by other chemicals^16^, but the mechanism of action of these compounds was unknown. In addition, the **089** HIPHOP profile clusters separately from the profiles of classical antifungals, confirming that this molecule acts through different mechanisms and does not trigger a response similar to the one induced by known antifungals in yeast cells. The **089** HIPHOP profile was similar to the HIPHOP profiles induced by two compounds (SGTC_1882 and SGTC_10) known to target the *RER2* gene, involved in dolichol biosynthesis. Dolichol is one of the building blocks of membrane structure, hence, a defect in the synthesis of this acid, such as that induced either by the deletion of the *RER2* gene^31^ or by its inhibition caused by treatments with SGTC_1882 and SGTC_10^16^, can trigger a downstream modification of cell morphology similar to that observed during treatment with **089**. Concerning the other treatments clustering with **089**, none of them was identified to have a specific target by Lee and colleagues^16^, probably because the Lee study was carried out on a smaller number of heterozygous deletion strains (only those deleted in essential genes) compared to the comprehensive pool used in the present work. It is thus possible that these compounds act on targets similar to that of **089**, whose target, *SWE1*, was not included in Lee and co-workers’ heterozygous deletion collection. In general, **089** shows a HIPHOP profile different from common antfungals including several azoles, amphotericin and caspofungin. Changes in fungal cell morphology and hyphae formation that reveal fungal components to immune receptors are crucial for the ability of the host immune system to respond to the pathogen. Indeed, fungal cells treated with **089** appear to be recognized and bound by immune cells at higher frequency than untreated cells. The increased binding by dendritic cells could be the result of either the inability of treated *Candida* spp. and *A. fumigatus* cells to initiate hyphal growth or the cell wall re-arrangement caused by **089**. However, we observed that the binding of dendritic cells to *S. cerevisiae* cells was not improved after the treatment with **089**. The *S. cerevisiae* interaction with dendritic cells has been shown to be mediated by the exposure of specific cell wall components, rather than by the yeast morphology^32^. Hence, these results suggest that the inability to initiate hyphal growth is the main factor responsible for the increased recognition of *Candida* spp. and *A. fumigatus* cells. Several pathogenic fungi (i.e. *Candida* spp. and *Aspergillus* spp.) switch their phenotype to invade the host tissue, evade the host immune response and shift it towards anti-inflammatory response^28^, thus **089** could represent a promising compound to boost the immune response or synergistically act with the host immune response in eradicating fungal infection.

## Methods

### Strains used in this study

Yeast and fungal strains and the mammalian cell line used in this study are listed in Table 1. The *Saccharomyces cerevisiae* heterozygous and homozygous diploid barcoded deletion collections were generated in the BY4742 genetic background (Invitrogen). Transcriptional analyses on *S. cerevisiae* were carried out on the BY4742 laboratory strain. The validation of the **089** target was carried out on single strain cultures of the BY4741 strain deleted in either the *HSL7*, the *SWE1* or the *RNY1* genes, which were knocked out through replacement with the KANMX4 cassette conferring resistance to geneticin (Invitrogen). As reference for DNA content quantification through flow-cytometry, the haploid BY4742 and the diploid BY4742xW303 strains were used. The *Candida albicans* SC5314 (ATCC MYA-2876) reference strain, the *C. glabrata* YUC22 (a clinical isolate) were used to evaluate the **089** MIC in pathogenic yeasts (Table 1). Three strains of *Aspergillus fumigatus* from the Bowyer lab collection were used to evaluate the **089** effect on the pathogenic fungus (Table 1): A1160, a reference strain (akuB (KU80)-delta pyrG1 MAT1-1); CYP51, an azole-resistant strain derived from the reference A1160 strain by deleting the cyp51A gene ((cyp51A)-delta akuB (KU80)-delta pyrG1 MAT1-1); RFLS58, a clinical isolate resistant to azoles. To evaluate the **089** cytotoxicity on mammalian cells, the K562 (ATCC CCL-243) human cell line was used (Table 1).

### Growth media and conditions

The *S. cerevisiae* and *Candida* spp. strains were grown and treated at 27 °C with shaking in YP (1% w/v Yeast Extract, 2% w/v Peptone) supplemented with 2% w/v glucose. Before treatments, yeast cells were grown over night in the same medium and environmental conditions as the treatment experiment. Treatments with the compound (or with DMSO as control) were carried out on cells inoculated at 5 × 10^6^ cells/ml concentration in YPD and treated in shaking at 27 °C.

*Aspergillus fumigatus* was grown on Sabouraud agar (1% w/v Peptone, 4% w/v glucose, 2% w/v agarose), at 37 °C. Conidia were collected from Sabouraud agar cultures using PBS + 0.1% Tween, avoiding collection of mycelial fragments and hyphae. The sample was filtered with a sterile filter with a pore diameter of 11 µm to remove hyphal fragments. Treatments with the compound were carried out in MOPS-buffered liquid RPMI 1640 medium (Euroclone) supplemented with 2% w/v glucose. The K562 cell line was grown in RPMI 1640 (Euroclone) supplemented with glutamine (Sigma Aldrich), penicillin and streptomycin (Sigma Aldrich), and 10% heat-inactivated fetal calf serum (Hyclone). The compound **089** ((7R)-3-benzhydryl-2-oxo-5-phenyl-6,8-dioxa-3-aza-bicycle[3.2.1]octane-7-carboxyl-ethylamide, MW = 442.51)^15^ was diluted in DMSO (DiMethyl SulfOxide) and added to the medium at the chosen concentration.

### Evaluation of the **089** effects on pathogenic yeasts and fungi

The *Candida* spp. strains were grown overnight in YPD medium and then inoculated in fresh medium at a final density of 5 × 10^6^ cells/ml. The **089** compound was added to the medium at various concentrations (0.3 mM, 0.2 mM, 0.1 mM), DMSO was used as control. After 4 hours of treatment at 27 °C with shaking, the *Candida* spp. cells survival was evaluated with the Live/dead® Yeast Viability Kit (Life technologies), as indicated by the manufacturer. *Candida* spp. cells CFU% (Colony Forming Units percentage) was evaluated by plating in YPD agar 100 treated live yeast cells and counting colonies formed after at least 4 days. *A. fumigatus* treatments were performed following the EUCAST protocol^33^. *A. fumigatus* spores were collected from solid Sabouraud cultures using PBS + 0.1% Tween, avoiding mycelium fragments and hyphae collection. 2.5 × 10^5^ spores/ml were inoculated in MOPS-buffered RPMI 1640 medium (Euroclone) supplemented with 2% w/v glucose. The effects of serial dilution of **089** ranging from 1 mM to 25 µM were assessed in 96-well plates using DMSO as control. Cultures were microscopically inspected after 48 hours of static growth at 37 °C. The treatment’s effects were read visually by recording the degree of growth for each well. The minimum effective concentration (MEC), defined as the lowest concentration of drug that results in macroscopic changes in filamentous growth to microcolonies or granular growth when compared with growth control wells, was evaluated for each *A. fumigatus* strain. For graphical reporting, the effect was scored as follows: 0 if all the cells were dead, 1 if no effects were observed compared to the control; between 0.25 and 0.75 for increasing hyphae formation inhibition.

### HIP HOP analyses

Both the homozygous and heterozygous barcoded-deletion pools (Table 1) were thawed and grown in YPD at 27 °C for 5 generations to allow cells to recover from freezing. Cells were then inoculated in 6 wells plates at 0.062 OD_600_ in YPD supplemented with 1% DMSO or 0.2 mM **089** (a sub-lethal compound concentration). Cells kept grown at 28 °C with shaking. Every five generations, 600 µl of the cultures were harvested and re-suspended with the same volume of YPD supplemented with the appropriate volume of DMSO or drug. After the 20^th^ generation, cells were harvested, and genomic DNAs were extracted using QIAamp DNA Mini Kit (Qiagen) following manufacturer’s instructions. Three independent biological replicates were carried out for each treatment. Barcodes amplification was carried out as described in Smith *et al*.^34^ and detailed in Fig. S9. Three independent replicates were carried out for each treatment. Raw data were normalized and HIPHOP profiles were identified as previously described^16^. To quantify the sensitivity of the deletion strains to the compound, we calculated for every deletion strain a robust Z score (Fitness Defect score, FD), standardized to allow the comparison of different profiles^16^. Aiming at this, the log2ratio was calculated for each strain as in equation [1].

1 $$\mathrm{log}\,2{{\rm{ratio}}}_{i,j}=\frac{{{\rm{reads}}}_{i,ctrl}}{{{\rm{reads}}}_{i,j}}$$

where reads_*i,ctrl*_ is the number of reads obtained for the *i*^*th*^ deletion strain in the control (*ctrl*) treatment and reads_*i,j*_ is the number of reads obtained for the *i*^*th*^ deletion strain in the treatment *j*.

Then, the FDs were calculated as in equation [2].

2 $${{\rm{F}}{\rm{D}}}_{i,j}=media{n}_{R}[\frac{{{\rm{l}}{\rm{o}}{\rm{g}}2{\rm{r}}{\rm{a}}{\rm{t}}{\rm{i}}{\rm{o}}}_{i,j}-median({{\rm{l}}{\rm{o}}{\rm{g}}2{\rm{r}}{\rm{a}}{\rm{t}}{\rm{i}}{\rm{o}}}_{j})}{MAD({{\rm{l}}{\rm{o}}{\rm{g}}2{\rm{r}}{\rm{a}}{\rm{t}}{\rm{i}}{\rm{o}}}_{j})}]$$

where *median*_*R*_ is the median among biological replicates and *MAD* is the Mean Absolute Deviation.

The concept of “clearance” was defined by Lee *et al*.^16^ and identifies hits that are substantially more significant than all other hits. After ordering the strains according to their FDs, the clearance is calculated as the difference of the FD of a given strain and of the closest strains. To identify strains significantly differing between the control and the treatment samples, we assumed that the FD scores followed a normal distribution in a screen and we calculated the probability that the given strain is an outlier in this distribution (one-sided p-value). Pathway analysis on HIP and HOP profiles was carried out by applying the Fisher’s exact test with EuGene analyzer on the list of significant strains^20^. Read counts of identified barcodes for each experiment were submitted to Array Express (E-SYBR-6) with the following sample names. **089**_HIP: B722, B726, B730; DMSO_HIP: B721, B725, B729; **089**_HOP: B695, B699, B703; DMSO_HIP: B692, B696, B700.

### HIP HOP meta-analysis

As an external dataset, the HIPHOP profiles produced by Lee and co-workers were downloaded from http://chemogenomics.pharmacy.ubc.ca/HIPHOP/16. The HIPHOP profiles were composed by strains deleted in essential genes in the heterozygous background and by strains deleted in non-essential genes in the homozygous background. Since our pools were composed by higher numbers of strains, we sub - divided them to make the profiles comparable. Then, the co-inhibition was calculated as described by Lee *et al*.^16^. The HIPHOP profiles are composed by significant FD scores (FD scores with p-values > 0.05 were replaced with 0), then the co-inhibition was calculated as the Pearson correlation between every couples of profiles. The significance of the correlation was estimated by using the Student’s *t*-distribution method. The clustering of profiles was then obtained by applying the Ward.D method of the *hclust* r function on the distances described as (1-coinhibition)^35^. Sub-clustering was identified in the obtained dendrogram using a dynamic branch cutting method by using the *cutreeDynamic* function of the dynamicTreeCut r package with the non-default parameter settings minimum cluster size = 3 and deepSplit = 4^36^.

### Microarray transcriptional analysis

BY4742 *S. cerevisiae* cells were grown overnight in YPD medium. The day after, 1 × 10^9^ cells were inoculated in 10 ml of fresh YPD supplemented with 0.2 mM **089** (sub-lethal concentration) or with DMSO. Cell cultures were incubated at 28 °C with shaking for 4 hours, then nitrogen frozen. RNA extraction was carried out as previously described^15^. Three independent biological replicates were carried out for each treatment. For hybridization onto Agilent 015072 Yeast Oligo Microarray 4 × 44 K 60mer oligonucleotide arrays (G2519F, Agilent), 300ng of total RNA were processed with the Agilent QuickAmp Labeling Kit, according to the manufacturer’s instructions. Fluorescent cDNA bound to the microarray was detected with a GenePix 4000B microarray scanner (Axon Instruments), using the GenePixPro6.1 software package to quantify microarray fluorescence. Data were normalized using the limma R package^37^ and DEGs (Differentially Expressed Genes) were identified with the RankProduct R package^38^. Raw and normalized data were submitted to GEO (GSE42418).

### Target validation – **089** effects on single cultures of *S. cerevisiae* deletion strains

Pure cultures of the heterozygous strains deleted in genes selected as potential **089** targets were grown in an overnight pre-culture in liquid YPD. Then, 10^5^ cells/ml were inoculated in 96-well plates in fresh YPD supplemented with either various concentrations of **089** (0.1 mM, 0.2 mM, 0.3 mM, 0.4 mM, 0.5 mM) or a corresponding volume of DMSO. The Optical Density (OD) was then measured every two hours. The percentage of growth was calculated as the difference of the treated culture’s OD and the control culture’s OD at the stationary phase (24 hours after the inoculum) divided by the control culture’s OD. Mann-Whitney test was carried out searching for significant differences between controls and treatments.

### Target validation – quantification of DNA content in *S. cerevisiae*

The cell cycle of BY4742 cells treated for 4 hours with 0.3 mM **089** at 28 °C with shaking was assessed by DNA content determination using Propidium Iodide (PI) staining and flow cytometry as previously described^39^. Control cells were treated with equal volumes of DMSO. As reference, Nitrogen starved haploid BY4742 cells (1 N) or BY4742xW303 diploid cells (2 N) at the exponential growing phase were used^40^. Fixation and PI staining were carried out as previously described^41^. Events were sub-selected (gated) when showing similar forward scattering (proportional to the cell size) and side scattering (proportional to cell complexity) values similar to untreated reference yeast cultures. In addition, cells were further selected according to the distribution of events in the plot of pulse area versus pulse width, allowing the elimination of overlapping doublet events, as previously described^42^.

### Target validation – quantification of total cell lipid in *S. cerevisiae*

To quantify the total amounts of ceramides and fatty acids, cells treated for 24 h with 0.3 mM **089** or with DMSO as control were pelleted, washed with water, Nitrogen-frozen then dried. The quantification was carried out in three independent biological replicates. Cells were subjected to lipid and fatty acids extraction as previously described^43^. Briefly, 2 g harvested cells were mixed with 1 ml methanol. Cells were disrupted by adding 2 g of glass beads and vigorously shaking for 4 periods of 30 s with 30 s cooling in dry ice intervals. 2 ml chloroform were added to the supernatant and the suspension was stirred for 1 h at room temperature. The organic phase was then transferred in a new tube where 1 ml of 0.034% MgCl_2_ solution was added. The mix was stirred for 10 min, then the aqueous phase was removed, and the organic phase was washed with 1 ml of 2 N KCl/methanol (4:1. v/v). The mix was centrifuged, and the resulting organic phase was washed with a solution of chloroform/methanol/water (3:48:47; per volume). The mix was centrifuged, and the solvent of the organic phase was evaporated in a speed vacuum at 55 °C. The lipid film was finally dissolved in 0.5 ml chloroform/methanol (2:1, v/v), tightly sealed and stored at −20 °C. Extracted lipids and fatty acids were spotted on a silica TLC plate, which was developed as previously described^44^. Briefly, polar lipids were separated by developing the chromatogram in a saturated chamber in chloroform-methanol/water (65:30:5, v:v:v). The solvent front was allowed to migrate 4 cm from the lower edge of the plate. The solvent was evaporated, and the plate was further developed in n-hexane-diethyl ether-acetic acid (80:20:1.5, v:v:v) to separate neutral lipids. Lipids were visualized by spraying plates with 20% ethanolic phosphomolibdic acid and heating at 180 °C for 10 min. Hence, lipids were visualized as dark spots at different distances from the lower edge of the plate. Palmitic acid, stearic acid and non-hydroxy fatty acid ceramide standards were purchased by SIGMA and used as reference to identify the lipid classes present in the samples (by comparing the distance from the lower edge of the plate). Lipids were quantified by measuring the area of the corresponding spot.

### Target validation – effects of **089** on the *S. cerevisiae* cell wall organization

To further validate the effects of **089** on the *S. cerevisiae* cell wall composition, the chitin distribution in the cell wall was evaluated by means of staining with calcofluor white. BY4742 cells from an over-night culture were inoculated (5*10^6^ cells/ml) in fresh YPD medium supplemented with 0.3 mM **089** (lethal concentration) or with DMSO (control). After four hours treatment, cells were washed with sterile water, the pellet was dissolved in 15 µl of sterile water and the cells were stained with calcofluor white (0.1 µg/ml). After one-minute incubation, cell membranes were visualized at a 100× magnification. Three independent biological replicates were carried out. The images of 10 randomly selected fields per replicate were captured.

### Assessment of the effects of anti-fungal combinations

To further explore the effect of the **089** compound, its effect on the survival of the laboratory *S. cerevisiae* strain BY4742 was evaluated in combination with antifungals modifying the yeast cell membrane. Aiming at this, the effects of all the combinations of four **089** concentrations (0.3 mM. 0.2 mM, 0.1 mM and 0.05 mM) and four concentrations of compounds impairing the cell membrane or wall composition were evaluated. The tested known compounds were: caspofungin (an echinocandin, inhibiting the β-glucan synthase), clotrimazole (an azole, inhibiting the biosynthesis of ergosterols), amphotericin B (acting by binding chitin and forming pores membrane on the cell membrane), and calcofluor white (binding the chitin). The tested concentrations included the MIC for *S. cerevisiae* and three lower concentrations. In particular, the tested concentrations were (for each compound, the highest one is the MIC): 0.252 µg/ml, 0.126 µg/ml, 0.063 µg/ml, and 0.0315 µg/ml for caspofungin; 3 µg/ml, 1.5 µg/ml, 0.75 µg/ml, and 0.375 µg/ml for clotrimazole; 1 µg/ml, 0.5 µg/ml, 0.25 µg/ml, and 0.125 µg/ml for amphotericin B; 25 µg/ml, 12.5 µg/ml, 6.25 µg/ml, and 3.125 µg/ml for calcofluor white. Cells from an over-night culture of the BY4742 strain were inoculated in fresh YPD medium supplemented with the compounds at various combinations or with DMSO as control. Each test was carried out in three independent biological replicates. After 24 hours treatment at 28 °C with shaking, the Optical Density of cultures was measured, and the survival percentage was calculated as in equation [3].

3 $${\rm{survival}}\, \% =\frac{{{\rm{OD}}}_{{\rm{t}}}\times 100}{{{\rm{OD}}}_{c}}$$

where OD_t_ is the OD of the treated culture and OD_c_ is the OD of the control culture. Significant differences were searched between the effect of each treatment and the effect of the lethal **089** concentration (0.3 mM) by mean of 2-tailed t-test. p-values were FDR corrected.

### *Aspergillus fumigatus* RNA sequencing and analysis

*A. fumigatus* conidia were collected from solid Sabouraud cultures using PBS + 0.1% Tween, avoiding collection of mycelium fragments and hyphae. Conidia were then inoculated in MOPS buffered RPMI 1640 medium + 2% glucose at 2.5 × 10^5^ conidia/ml concentration supplemented with either 0.1 mM **089** molecule or an equal volume of DMSO. After 4 hours of growth in static at 37 °C, RNA was extracted from liquid nitrogen powder grounded samples with Trizol. Each treatment was carried out in three independent biological replicates. For each sample, one paired-end mRNA library with approximately 150 bp insert size was prepared using the TruSeq RNA Sample Preparation Kit v2 (Illumina). Libraries were sequenced on Illumina HiSeq 2000 lanes using 2 × 75 bp reads. The cluster generation kit was TruSeq PE Cluster Kit v3-cBot-HS, and the sequencing kit was TruSeq SBS Kit v3-HS. More than 40 million reads were generated for each sample. Raw reads were filtered and trimmed by using the IlluQC and TrimmingReads tools of the NGS QC Toolkit package^45^. The filtering process was carried out with the default options. The trimming was carried out with mild thresholds namely -q 5 -n 25. Alignment has been done by using tophat2 with the *Aspergillus fumigatus* A1160 reference genome sequence downloaded from NCBI (download date: 4 Nov 2016). The FPKMs (Fragments per Kilobase of transcript per Million mapped reads) were calculated to determine the expression level of each gene. The differential expression analysis was performed using the cuffdiff tool of the cufflinks package^45^. DEGs were identified based on a false discovery rate (FDR) < 0.05. Gene Ontology (GO) enrichment analysis was carried out on DEGs lists by using BiNGO^46^ and visualized with Cytoscape^47^. We compared the transcriptional profile of the **089** treatment with the transcriptional profiles of *A. fumigatus* cells at different stages of the asexual cell cycle^27^. We then calculated the Manhattan distance among all the samples and genes and drew the cluster with the ward.D method. Read counts of identified sequences for each experiment are available in the ArrayExpress database (www.ebi.ac.uk/arrayexpress) under accession number E-MTAB-5309.

### Target validation – effects on *Aspergillus fumigatus* isotropic growth rate and nuclear division

The A1160 *A. fumigatus* strain was used to assess the effect of **089** on the fungal isotropic growth rate and nuclear division. The internalization of **089** drug granules within the fungal conidia/ germling was evaluated by observing microscopic auto fluorescent drug particles ~30 min post treatment. The localization of drug granules was assessed before and after washing twice the treated cells with PBS.

The volume and area of conidia treated with 0.4 mM **089** or with DMSO as control were measured using Imaris software (Bitplane, UK) of a confocal acquisition time lapse over 6 hours obtained using a Leica SP10 microscope.

To count the number of nuclei of germinated *A. fumigatus* conidia, an A1160 *A. fumigatus* strain expressing the labelled Histone 1 protein (H1-sGFP) was used. Conidia were grown in Sabouraud medium for 6 hours at 37 °C, then treated with 0.4 mM **089** or with DMSO as control. Images were captured every hour over a time course of 6 hours.

### Dendritic cells (DC) binding assay

The experimental plan was approved by the local Ethical Committee of the Careggi hospital (Florence, Italy, Ref. n. 87/10), and informed consent was obtained from all the healthy donors. All methods were performed in accordance with the relevant guidelines and regulations. Peripheral Blood Mononucleated Cells (PBMCs) were isolated from buffy coat blood samples from healthy volunteers by Ficoll-Hypaque density gradient centrifugation. Monocytes were isolated from low density PBMCs by magnetic enrichment with anti-CD14 beads (Miltenyi Biotec). After isolation, monocytes were cultured in RPMI 1640 supplemented with glutamine, penicillin and streptomycin, and 10% heat-inactivated fetal calf serum in the presence of GM-CSF (800 U/ml, Gentaur) and recombinant IL-4 (1000 U/ml, Gentaur) for 6 days to allow dendritic cells (DCs) differentiation^48^. *S. cerevisiae*, *C. albicans* and *A. fumigatus* cells were treated for 4 hours with sub-lethal concentrations of the **089** compound (0.2 mM). *S. cerevisiae* and *C. albicans* cells were treated in YPD at 28 °C with shaking, A. fumigatus cells were treated in MOPS-buffered RPMI medium at 37 °C in static. After the treatment, cells were washed with sterile water and the labelling of *S. cerevisiae*, *C. albicans* and *A. fumigatus* was performed before exposure to DCs as previously described^49^. FITC-labelled fungi were added in a fungal cell:DC ratio of 5:1. After 30 minutes of incubation at 37 °C, cell-fungi conjugates were analysed by flow cytometry. The binding of DCs to fungi was measured by flow cytometry using a FACS Calibur instrument (BD Biosciences). DCs were labelled with anti-CD11c-PE to discriminate cells binding FITC-labelled yeast/spores particles from yeast/spores aggregates. Mann-Whitney test was used to evaluate the statistical significance of the results by comparison between treated and untreated strains.

### Cytotoxicity on mammalian cells

K562 cells were grown in RPMI 1640 (Euroclone) supplemented with glutamine (Sigma), penicillin and streptomycin (SIGMA), and 10% heat-inactivated fetal calf serum (Hyclone). For toxicity assays, 3*10^5^ cells/well were treated for 24 hours in 6-wells plates. Treatments were carried out with various concentrations of **089** (from 0.1 mM to 0.4 mM), ketoconazole (from 2.5 μM to 0.1 mM), or DMSO as control. Cell death induced by the treatments was assessed by the trypan blue exclusion technique. Survival percentage was evaluated as the number of live cells after the treatment with respect to the number of live cells after control treatment (cells grown without either the antifungals or DMSO). ANOVA statistics were used to evaluate differences among treatment effects.

### Comparison of *SWE1* sequences in yeasts and fungi

To support the possibility that the same gene is targeted by **089** in different yeasts and fungi, we compared the *S. cerevisiae* Swe1p sequence with the available sequences of homologues in other yeasts and fungi. The *S. cerevisiae* Swe1p sequence was downloaded from the SGD website (date of access 26 August 2017)^50^. Ortholog sequences in fungi were retrieved from the OrthoDB database, using the *S. cerevisiae* Swe1p sequence as query^51^. The so-obtained sequences were then aligned with Muscle. The Neighbor-joining tree was inferred from distances calculated as the number of substitutions as a proportion of the length of the alignment (excluding gaps) with the Simple Phylogeny tool at EMBL-EBI^52^ and drawn with figtree (http://tree.bio.ed.ac.uk/).

## Electronic supplementary material

Supplementary information

Movie S1

Movie S2

Movie S3

Movie S4

Movie S5

Movie S6

Movie S7

Movie S8

Table S1

Table S2

Table S3

Table S4

Table S5

Table S6
