# Supplementary material for: Deciphering the mechanism of action of 089, a compound impairing the fungal cell cycle
Source: Sci Rep. 2018 Apr 13;8:5964. doi: 10.1038/s41598-018-24341-y (PMC5899093; doi:10.1038/s41598-018-24341-y)
Supplement: Supplementary file 1 — Supplementary information [file 41598_2018_24341_MOESM1_ESM.pdf]

Supplementary Information for

Deciphering the mechanism of action of 089, a compound impairing the fungal cell cycle

Irene Stefanini, Lisa Rizzetto, Damariz Rivero, Silvia Carbonell, Marta Gut, Simon Heath, Ivo G. Gut, Andrea Trabocchi, Antonio Guarna, Nagwa Ben Ghazzi, Paul Bowyer, Misha Kapushesky, Duccio Cavalieri

This file includes:

Figure S1: Schematic representation of the strategy adopted to identify the mechanism of action of the new anti-fungal drug.

Figure S2: HIPHOP meta-analysis.

Figure S3: Effects on *S. cerevisiae* growth of **089** in combination with compounds known to perturb the cell wall.

Figure S4: Effects of **089** on the *S. cerevisiae* cell wall

Figure S5: Effect of *A. fumigatus* strains treatment with **089**.

Figure S6: Phylogenetic tree of Swe1p orthologs in fungi

Figure S7: assessment of the **089** cytotoxicity on the human leukemic K562 cell line

Figure S8: schematic representation of the barcodes sequencing approach. PCR amplification was carried out before sequencing the deletant pool barcodes

Table S7: primers used for barcodes amplification.

Other Supplementary Materials for this manuscript:

Table S1: Fitness Defect scores calculated for heterozygous deletion strains

Table S2: Fitness Defect scores calculated for homozygous deletion strains and pathways differentially represented in HOP profile as calculated with EuGene Analyzer (Beltrame et al. 2009)

Table S3: significant FC of expressed genes as quantified through expression microarray and pathways differentially expressed after the treatment of *S. cerevisiae* cells with **089** as calculated with EuGene Analyzer (Beltrame et al. 2009).

Table S4: HIPHOP profiles of the Lee database (Lee 2013) clustering with the HIPHOP profile induced by 089.

Table S5: Differentially Expressed Genes after the treatment of A1160 *Aspergillus fumigatus* conidia with

a sub-lethal concentration of **089**.

Table S6: Gene Ontology terms enriched in the list of DEGs found after the treatment of *Aspergillus fumigatus* conidia with **089**.

Supplementary videos:

Supplementary Movies S1-S4: video capturing the effect of **089** on *Aspergillus fumigatus* germination. *A. fumigatus* A1160 conidia were grown in Sabouraud medium for 6 hours at 37° C, then treated with 0.4 mM **089**. Images were captured every hour over a time course of 6 hours. *A. fumigatus* nuclei are visualized in green as the strain has been genetically modified to express a GFP labeled Histone 1 protein (H1-sGFP). Each video (S1-S4) reproduces a biological independent replicate of the experiment.

Supplementary Movies S5-S8: video capturing the effect of DMSO on *Aspergillus fumigatus* germination. *A. fumigatus* A1160 conidia were grown in Sabouraud medium for 6 hours at 37° C, then treated with DMSO (control treatment). Images were captured every hour over a time course of 6 hours. *A. fumigatus* nuclei are visualized in green as the strain has been genetically modified to express a GFP labeled Histone 1 protein (H1-sGFP). Each video (S5-S8) reproduces a biological independent replicate of the experiment.

Figure S1

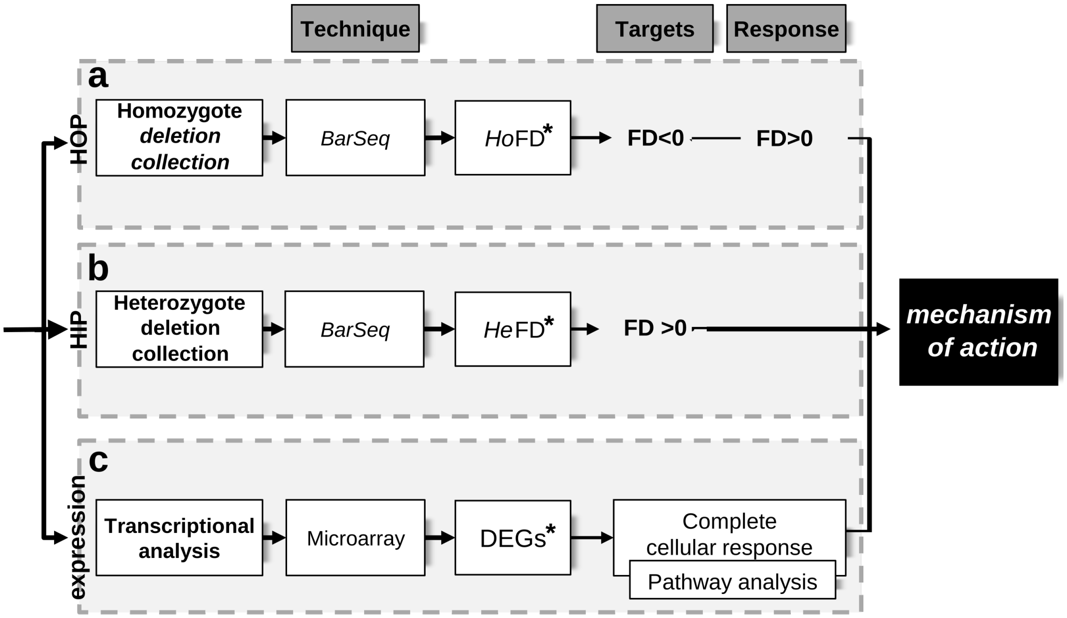

Schematic representation of the strategy adopted to identify the mechanism of action of the new anti-fungal drug. a- HOP: HOmozygous deletion Profiling, treatment of the homozygous barcoded deletion collection and identification of strains with significant Fitness Defect scores (*Ho*FD) through *BarSeq* (*Bar*coded *Seq*uencing)**.** *Ho*FD with FD<0 are related to the compound target. *HoFD* with FD>0 indicate the defense to the treatment. **b-** HIP: HaploInsufficiency Profiling, treatment of the heterozygous barcoded deletion collection and identification of strains with significant Fitness Defect scores (*He*FD) through *BarSeq*. *HeFD* with FD>0 are possible targets. **c-** Treatment of the wild-type laboratory strain BY4742 and identification of Differentially Expressed Genes (DEGs) by means of microarray transcriptional analysis. This procedure allows the profiling of the complete transcriptional status of the cell after the treatment. *=selected as significant, for details please refer to materials and methods.

Figure S2

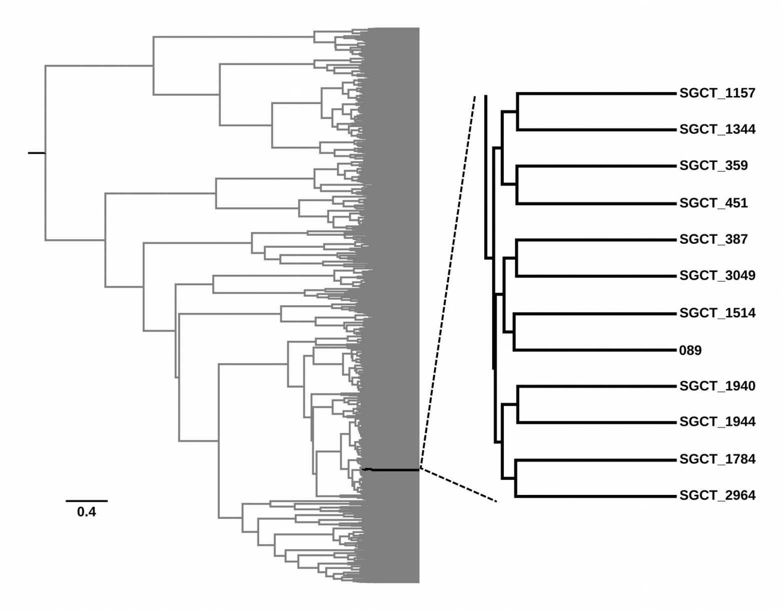

HIPHOP meta-analysis. Hierarchical clustering was carried out with the Ward method on the distance among samples HIPHOP calculated as 1-(Pearson r). In black the sub-cluster encompassing the **089** HIPHOP, identified through a dynamic branch cutting method. On the right magnification of the sub-cluster encompassing the **089** HIPHOP.

Figure S3

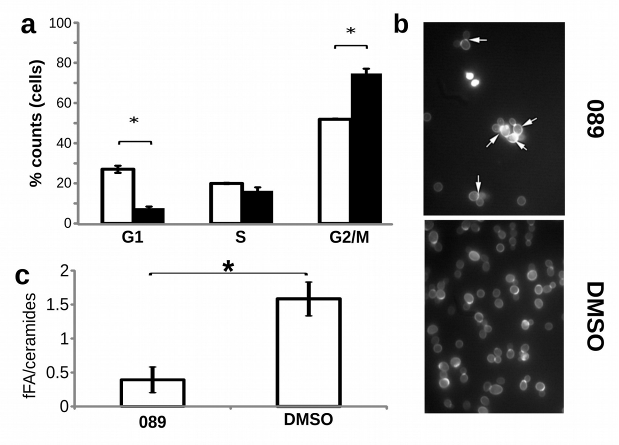

Phenotypic effects of Swe1p targeting by 089. a- 089 induces yeast cells to accumulate in the G2/M phase. The cell cycle of BY4742 cells treated for 4 hours with 0.3mM **089** (black bars) was assessed in comparison to the control (DMSO, white bars) by DNA content determination using Propidium Iodide (PI) staining and flow cytometry. Values: mean number of cell per DNA content evaluated by flow cytometry. n=3, error bars: SD, *2-tailed t-test $p<0.01$; **b-** Bud morphology is affected by the treatment with **089**. Cells were treated for 4 hours with 0.3M **089** or equal volumes of DMSO, then stained with Calcofluor white. Arrows: cells having multiple/abnormal buds; **c-** cells treated with **089** accumulate ceramides over free fatty acids (fFA), *= Wilcoxon $p<0.05$.

Figure S4

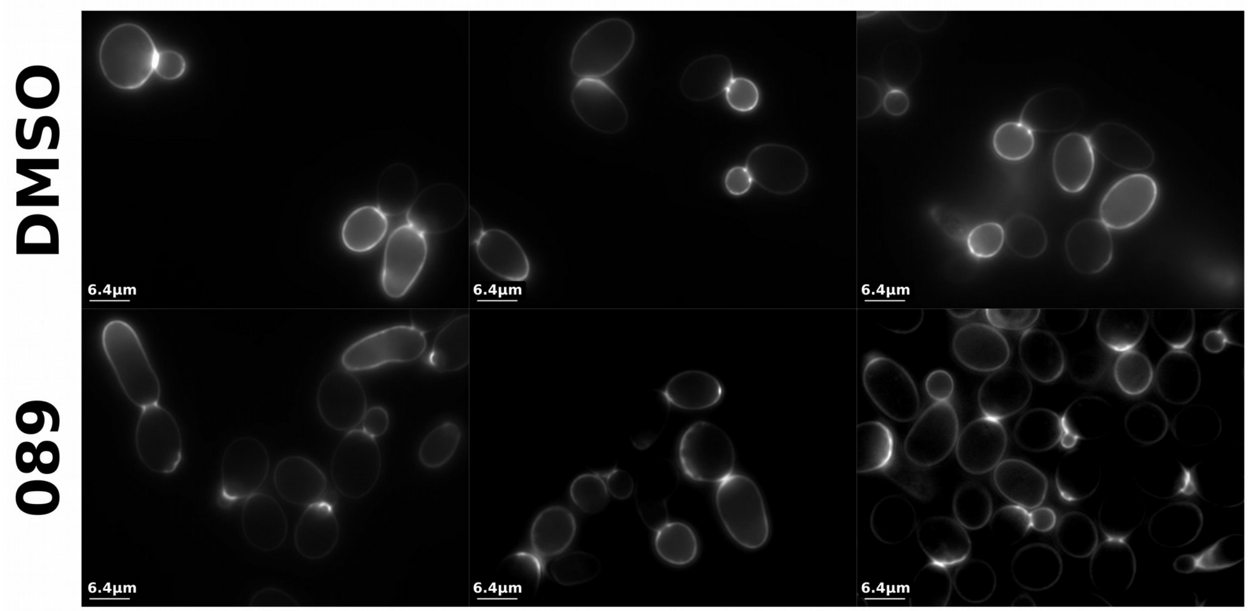

Effects of 089 on the *S. cerevisiae* cell wall. BY4742 cells were treated with 0.3mM **089** or DMSO for 4 hours in YPD with shacking at 28°C. After the treatment, cells were washed with sterile water and labeled with calcofluor white (0.1µg/ml) and visualized at a 100x magnification. Three independent biological replicates were carried out. Images were acquired for 10 randomly selected fields per replicate.

Figure S5

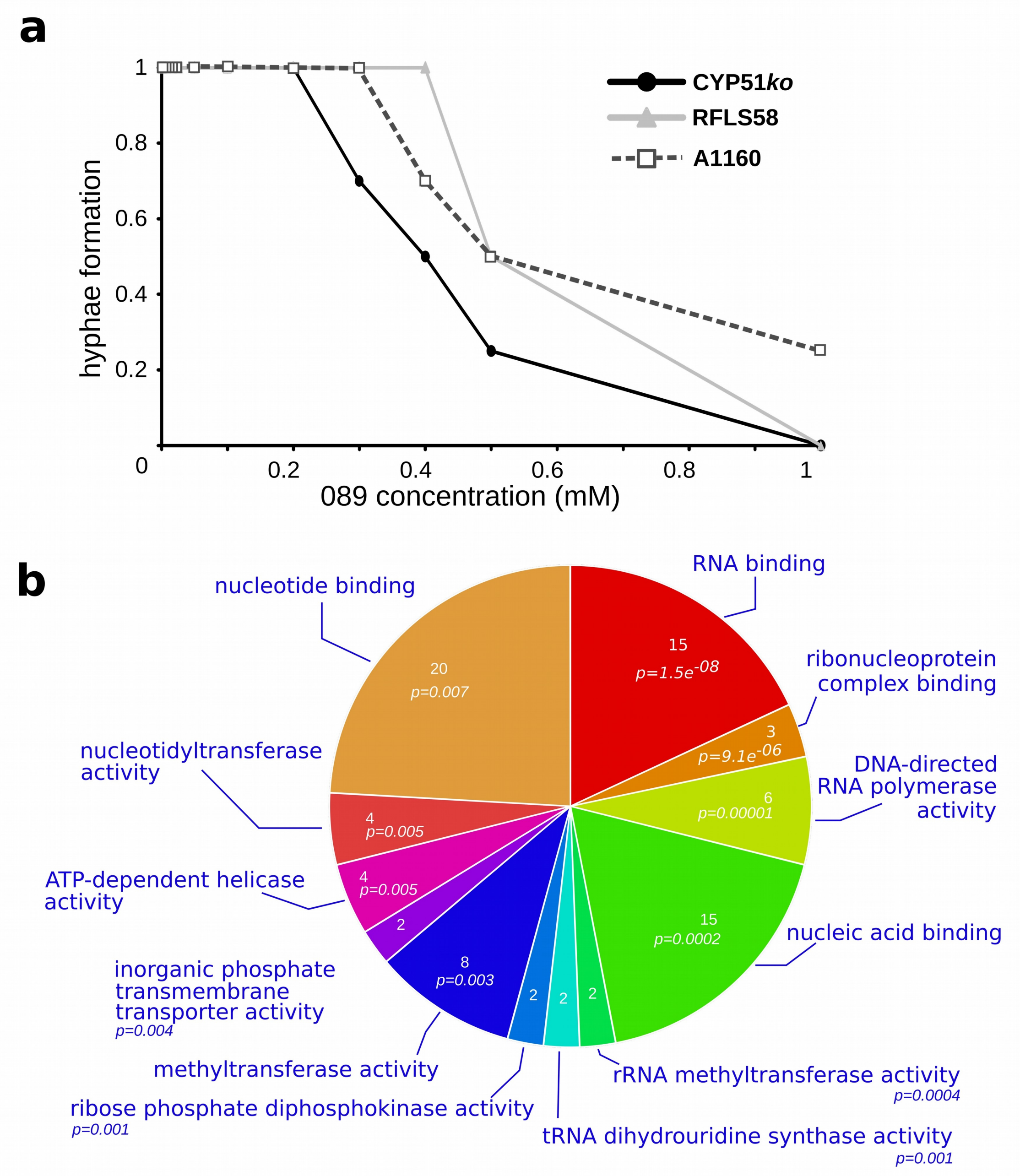

Effect of the treatment of A. fumigatus strains with 089. a- MIC determinations were performed following the EUCAST method. The effect reported in the y axis was scored as: cell death=0, hyphae formation inhibition=0.25-0.75, no effects=1. **b-** Gene Ontologies enriched in the list of genes overexpressed by *A. fumigatus* treated with 089 and overexpressed in Germinating conidia compared to hyphae but under-expressed in conidia compared to either germinating conidia or hyphae.

Figure S6

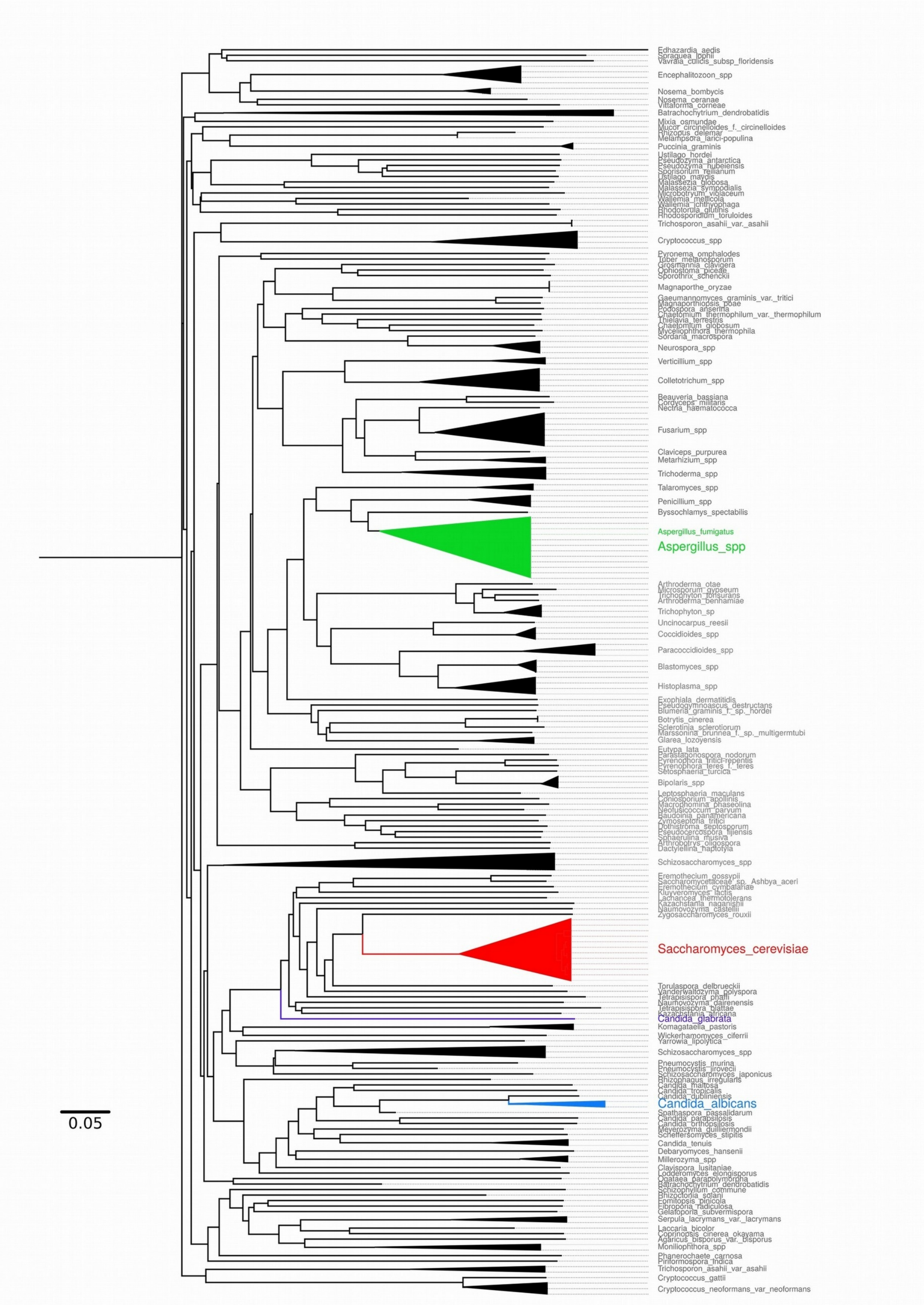

Phylogenetic tree of Swe1p orthologs in fungi. Ortholog sequences were obtained from the OrthoDB database (http://www.orthodb.org/), aligned with Muscle, clustered with the neighbor-joining algorithm and drawn the tree with figtree.

Figure S7

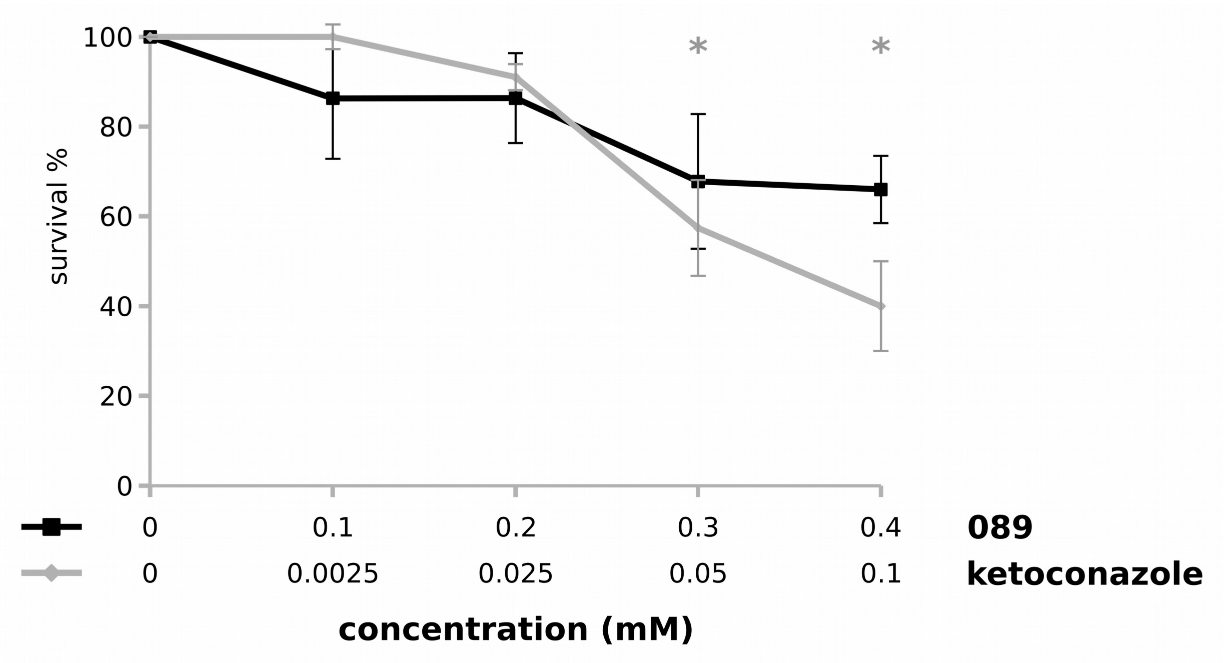

Assessment of the 089 cytotoxicity on the human leukemic K562 cells. Cells were grown in RPMI 1640 (Lonza) supplemented with glutamine (Sigma), penicillin and streptomycin (SIGMA), and 10% heat-inactivated fetal calf serum (Hyclone). For toxicity assay, 300000 cells/well were treated for 24 hours in 6-wells plates. Treatments were carried out with various concentrations of **089** (from 0.1mM to 0.4mM), ketoconazole (from 2.5μM to 0.1mM), or DMSO as control. Death induced by the treatments was assessed by the trypan blue exclusion technique. Survival percentage was evaluated as the number of live cells after the treatment with respect to the number of live cells after control treatment (cells grown without either the antifungals or DMSO). Measures are represented as means+SD. ANOVA statistics was performed to evaluate differences among treatment effects, *comparison with the control treatment, p-val<0.05. Only ketoconazole treatments at high concentrations are significantly reducing the survival of mammalian cells.

Each 20-mer uptag barcode was amplified with an unique forward primer (UpTag U1 Forward)(30), having the U1 sequence plus the sequence R2 required for cluster formation on the Illumina flowcell. Twelve reverse primers were specifically designed with PerlPrimer v1.1.19 Software (Copyright © 2003-2010 Owen Marshall) (**Figure S8**). The reverse composite primers contain sequences of the common barcode primers that will anneal upstream of U2 in a specific region of the kanamycin sequence, 12 different indexing tags of 6 nucleotides and the sequence R1 required for hybridization to the Illumina flowcell and for indexing tags reading (**Table S7**). After amplification, products were clustered in a single-end flow cell and sequenced on an Illumina GA2x – 36 bases for the read and 7 bases for the index. The adopted protocol was an adaptation of the method used by Smith (Smith et al.). Each PCR amplification was carried out in 50 µl, using 100 ng of sample, 0.5µM of each forward and reverse primer (Oligosynthese BioTeZ, Germany) and 2.5 U of Invitrogen Accuprime Pfx DNA polymerase (Cat. No 12344-024) with the following cycling conditions: 1 cycle at 95ºC for 3 min; and 25 amplification cycles (94ºC for 30 s, 55ºC for 30 s, 68ºC for 30 s); followed by 68ºC for 10 min. PCR products were then purified with Beckman Coulter SPRI beads (Cat. No. 082A63881) using a ratio of 1. After PCR purification, DNA was quantified with 7500 DNA chip on 2100 Bioanalyzer (Agilent). Equimolar amounts of PCR products were pooled and run on an Illumina GAIIx using an Illumina single read flowcell (Cat. No. GD-300-2001) with 36 sequencing cycles for the first read and 7 cycles for the index read (Cat. No. FC-104-5020). The UP-tag U2 seq primer (Oligosynthese BioTeZ, Germany) and the R1 Index Sequencing primer (Included in Cat. No. FC-104-5020 kit) were used for the first read and for the index read, respectively.

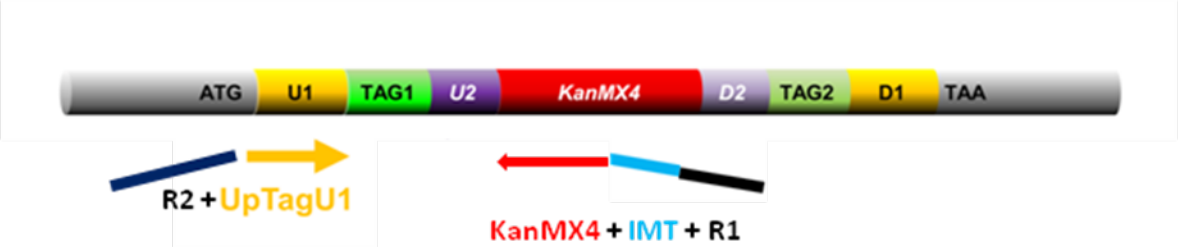

<u>Figure S8:</u>

schematic representation of the barcodes sequencing approach. PCR amplification was carried out before sequencing the deletant pool barcodes. The forward primer used for this purpose contains two different sequences: UpTagU1 and R2 (for cluster formation on the Illumina flowcell). The reverse primer is composed by three different sequences: KanMX4 (kanamicin specific sequence close to the U2 region), IMT (Internal Multiplexing Tag, required to allow postsequencing assignment of each amplicon to particular experimental conditions) and R1 (allows hybridization of amplicons to the Illumina flowcell and also contains the required sequence to read Illumina multiplexing tags).

Name	Sequence (5’- > 3’)
UpTag U1 Forward	CAA GCA GAA GAC GGC ATA CGA GCT CTT CCG ATC *TGA TGT CCA CGA GGT CTC T*
DownTag KanB Reverse IS-1	AAT GAT ACG GCG ACC ACC GAC ACT CTT TCC CTA CAC GAC GCT CTT CCG ATC T**AT CAC G***CT GCA GCG AGG AGC CGT AAT*
DownTag KanB Reverse IS-2	AAT GAT ACG GCG ACC ACC GAC ACT CTT TCC CTA CAC GAC GCT CTT CCG ATC T**CG ATG T***CT GCA GCG AGG AGC CGT AAT*
DownTag KanB Reverse IS-3	AAT GAT ACG GCG ACC ACC GAC ACT CTT TCC CTA CAC GAC GCT CTT CCG ATC T**TT AGG C***CT GCA GCG AGG AGC CGT AAT*
DownTag KanB Reverse IS-4	AAT GAT ACG GCG ACC ACC GAC ACT CTT TCC CTA CAC GAC GCT CTT CCG ATC T**TG ACC A***CT GCA GCG AGG AGC CGT AAT*
DownTag KanB Reverse IS-5	AAT GAT ACG GCG ACC ACC GAC ACT CTT TCC CTA CAC GAC GCT CTT CCG ATC T**AC AGT G***CT GCA GCG AGG AGC CGT AAT*
DownTag KanB Reverse IS-6	AAT GAT ACG GCG ACC ACC GAC ACT CTT TCC CTA CAC GAC GCT CTT CCG ATC T**GC CAA T***CT GCA GCG AGG AGC CGT AAT*
DownTag KanB Reverse IS-7	AAT GAT ACG GCG ACC ACC GAC ACT CTT TCC CTA CAC GAC GCT CTT CCG ATC T**CA GAT C***CT GCA GCG AGG AGC CGT AAT*
DownTag KanB Reverse IS-8	AAT GAT ACG GCG ACC ACC GAC ACT CTT TCC CTA CAC GAC GCT CTT CCG ATC T**AC TTG A***CT GCA GCG AGG AGC CGT AAT*
DownTag KanB Reverse IS-9	AAT GAT ACG GCG ACC ACC GAC ACT CTT TCC CTA CAC GAC GCT CTT CCG ATC T**GA TCA G***CT GCA GCG AGG AGC CGT AAT*
DownTag KanB Reverse IS-10	AAT GAT ACG GCG ACC ACC GAC ACT CTT TCC CTA CAC GAC GCT CTT CCG ATC T**TA GCT T***CT GCA GCG AGG AGC CGT AAT*
DownTag KanB Reverse IS-11	AAT GAT ACG GCG ACC ACC GAC ACT CTT TCC CTA CAC GAC GCT CTT CCG ATC T**GG CTA C***CT GCA GCG AGG AGC CGT AAT*
DownTag KanB Reverse IS-12	AAT GAT ACG GCG ACC ACC GAC ACT CTT TCC CTA CAC GAC GCT CTT CCG ATC T**CT TGT A***CT GCA GCG AGG AGC CGT AAT*
UP-tag U2 Seq	GTC GAC CTG CAG CGT ACG
R1 Index Sequencing	AC ACT CTT TCC CTA CAC GAC GCT CTT CCG ATC T

Table S7:

Primers used for barcodes amplification. The 5’ portion (underlined) of Forward primer contains the sequence R2 and the 3’ portion (italics) corresponds to the U1 sequence. The three regions of the 12 reverse composite primers, the portion 5’ (underlined), the internal portion (bold) and the 3’ portion (italic) correspond to R1, IMT and KanMX4 sequences, respectively (**Supplementary Figure S9**)
